# Structural and Functional Characterization of a Novel α-Conotoxin Mr1.7 from *Conus marmoreus* Targeting Neuronal nAChR α3β2, α9α10 and α6/α3β2β3 Subtypes

**DOI:** 10.3390/md13063259

**Published:** 2015-05-27

**Authors:** Shuo Wang, Cong Zhao, Zhuguo Liu, Xuesong Wang, Na Liu, Weihong Du, Qiuyun Dai

**Affiliations:** 1 Beijing Institute of Biotechnology, Beijing 100071, China; E-Mails: crosswang@gmail.com (S.W.); liuzhuguo@126.com (Z.L.); liuna1719@126.com (N.L.); 2 Department of Chemistry, Renmin University of China, Beijing 100872, China; E-Mails: zhaoc271@ruc.edu.cn (C.Z.); xuesong_wang@ruc.edu.cn (X.W.)

**Keywords:** neuronal nicotinic acetylcholine receptor, α-conotoxin Mr1.7, *Conus marmoreus*, selectivity, *N*-terminal sequence, structure-activity relationship

## Abstract

In the present study, we synthesized and, structurally and functionally characterized a novel α4/7-conotoxin Mr1.7 (PE**CC**THPA**C**HVSHPEL**C**-NH_2_), which was previously identified by cDNA libraries from *Conus marmoreus* in our lab. The NMR solution structure showed that Mr1.7 contained a 3_10_-helix from residues Pro^7^ to His^10^ and a type I β-turn from residues Pro^14^ to Cys^17^. Electrophysiological results showed that Mr1.7 selectively inhibited the α3β2, α9α10 and α6/α3β2β3 neuronal nicotinic acetylcholine receptors (nAChRs) with an IC_50_ of 53.1 nM, 185.7 nM and 284.2 nM, respectively, but showed no inhibitory activity on other nAChR subtypes. Further structure-activity studies of Mr1.7 demonstrated that the PE residues at the *N*-terminal sequence of Mr1.7 were important for modulating its selectivity, and the replacement of Glu^2^ by Ala resulted in a significant increase in potency and selectivity to the α3β2 nAChR. Furthermore, the substitution of Ser^12^ with Asn in the loop2 significantly increased the binding of Mr1.7 to α3β2, α3β4, α2β4 and α7 nAChR subtypes. Taken together, this work expanded our knowledge of selectivity and provided a new way to improve the potency and selectivity of inhibitors for nAChR subtypes.

## 1. Introduction

As ligand-gated ion channels, neuronal nicotinic acetylcholine receptors (nAChRs) are widely spread in the central and peripheral system [1]. nAChRs modulate the release of neurotransmitters, such as dopamine, norepinephrine, acetylcholine and γ-amino butyric acid [2], and they are involved in a variety of pathophysiologies, including chronic pain syndromes, epilepsy, Parkinson’s and Alzheimer’s [3,4,5,6]. To date, eight α and three β subunits (α2–α7, α9, α10, β2–β4) of nAChRs have been identified from mammalian neuronal cells [7]. They can form homopentamers (such as α7) and heteropentamers (such as α3β2, α3β4, α2β4, α4β2, α6β2β3, α2β2, and α9α10 subtypes) [8]. The nAChR α3β2 subtype is expressed in the dorsal root ganglia and spinal cord, and it is involved in pain sensation [9,10]. nAChR α9α10 has been shown to be expressed on the cochlear outer hair cells, where it mediates the cholinergic efferent transmission [11]. Several α-conotoxins have been reported to possess potent analgesic activity. Among them, Vc1.1 and RgIA selectively target nAChR α9α10, and MII selectively targets nAChR α3β2 [12,13].

As small disulfide-rich peptides from the venom of Cone snail, conotoxins (CTX) are classified into several superfamilies, including A, B3, D, M, and J superfamily, and so on [14], based on their conserved signal sequence. α-CTXs belong to A-superfamily and selectively target nAChRs. They are usually composed of 12–18 amino acids with two disulfide bonds. According to the residue numbers of the inter cysteine loops (-CC-(loop1)-C-(loop2)-C-), they can be further divided into several subfamilies. For example, the α3/5-CTXs mainly target muscle nAChRs, while the α4/7-CTXs are specific inhibitors of neuronal nAChRs [9]. To date, most α-CTXs contain a Gly prior to the first Cys, and only a few α-CTXs contain an extended amino acid sequence except for Gly, such as GID [15], AnIB [16], LsIA [17] and MI [18]. Some α-CTXs have already become valuable neuropharmacological tools and drug leads [19,20].

In the present study, we synthesized and, structurally and functionally characterized a novel α4/7-conotoxin Mr1.7 (PE**CC**THPA**C**HVSHPEL**C**-NH_2_), which was previously identified by cDNA libraries from *Conus marmoreus* in our lab [21]. Mr1.7 belonged to the typical α4/7-CTXs and specifically inhibited nAChRs α3β2, α9 α10 and α6/α3β2β3 with an IC_50_ of 53.1 nM, 185.7 nM and 284.2 nM, respectively, but showed no inhibitory activity on other nAChR subtypes. This property was significantly different from the reported α-CTXs. Furthermore, we also investigated the structure-function relationship of Mr1.7. The results showed that the PE residues ahead of the *N*-terminal of Mr1.7 were important for modulating its selectivity, and the substitution of Ser^12^ with Asn in the loop2 significantly increased the binding of Mr1.7 to α3β2, α3β4, α2β4 and α7 nAChR subtypes. Taken together, our work expanded our knowledge of selectivity of α-CTXs and provided a new way to improve the potency and selectivity for nAChR subtypes.

## 2. Results

### 2.1. Chemical Identity of Synthetic Mr1.7 and Its Variants

**Figure 1:**
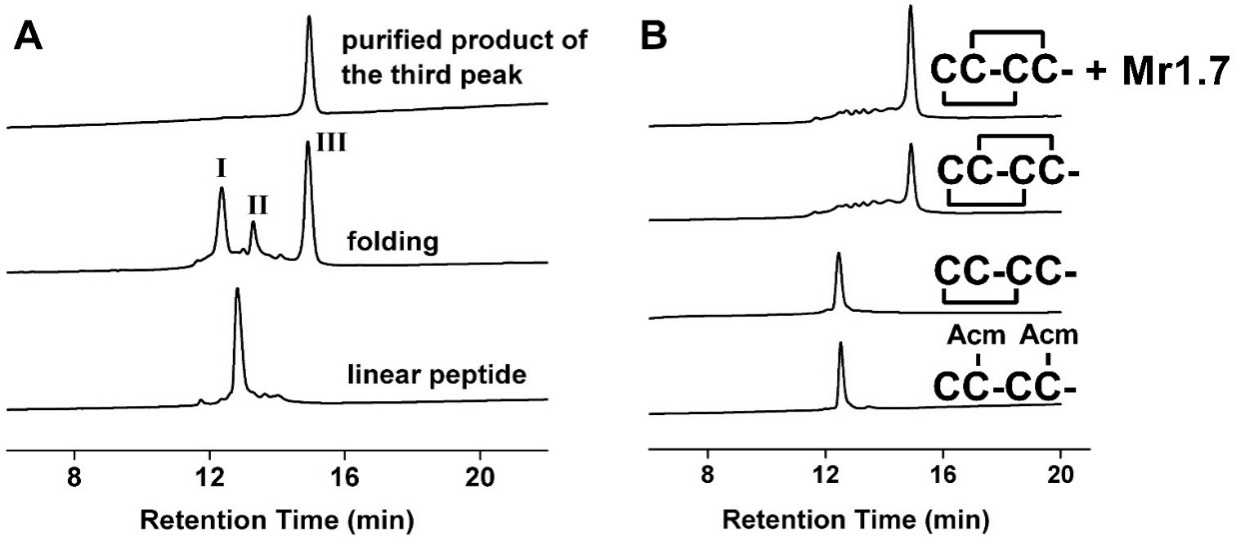
HPLC analyses of the folded products of linear Mr1.7 and its Acm derivatives. (**A**) One-step oxidative folding of Mr1.7. Traces from bottom to top: linear peptide; one-step oxidized products; and purified product of the third peak; (**B**) Determination of the disulfide bond connectivity of Mr1.7. Traces from bottom to top: linear peptide with Acm modification at Cys^2^ and Cys^4^; the primary oxidized product; the secondary oxidized product and co-elution of the two-step folding products and one-step folding products. Samples were applied onto a Calesil ODS-100 C18 column (4.6 mm × 250 mm) and eluted with a linear gradient of 0–1 min, 5%–10% B; 1–25 min, 10%–50% B; 25–28 min, 50%–95% B (B is acetonitrile (0.1% TFA)), at a flow rate of 1 mL/min, 214 nm.

Mr1.7 and its variants were synthesized and assessed by analytical reversed-phase HPLC (Figure 1A, Table 1). The molecular weight of all peptides ascertained by Ultraflex III TOF/TOF mass spectrometry (Bruker, Bremen, Germany) was consistent with the calculations (see Supplementary Table S1).

### 2.2. Disulfide Bridge Pattern of Mr1.7 Is I-III, II-IV

Figure 1B shows the HPLC analysis of the one-step and two-step folding experiments of Acm-protected linear peptides. The retention time of Mr1.7 synthesized by one-step was identical with that of Mr1.7 folded by two-step oxidation method, demonstrating that Mr1.7 possessed a disulfide connectivity (I-III, II-IV). Usually, the linear Mr1.7 variant folded one or two main products. The unity of disulfide bond connectivity of Mr1.7 variants was determined by CD spectra, the results showed that only one folding product had similar α-helical structure with Mr1.7 (Supplementary Figure S1), suggesting that the correct folding products had the same disulfide bond connectivity (I-III, II-IV).

**Table 1:** Amino acid sequences, activity and selectivity of α-CTX Mr1.7 and its variants. The cysteines and mutated residues are in boldface; the mutated residues are in italic face; numbers in parentheses are IC_50_s and 95% confidence intervals; and * *C*-terminal carboxamide.

α-CTX	Amino Acid Sequence	Targets (IC_50_, nM)	α3β4/α3β2	α2β4/α3β2	α7/α3β2	α9α10/α3β2
Mr1.7	PE**CC**THPA**C**HVSHPEL**C** *	α3β2 (53.1(48.0–58.8)), α9α10 (185.7(154.1–223.7)), α6/α3β2β3 (284.2(199.4–405.2)), α3β4 (>10,000), α7 (>10,000), α2β4 (>10,000), α2β2 (>10,000), α4β2 (>10,000), α4β4 (>10,000)	>188	>188	>188	3.5
RaaMr1.7	***R***PE**CC**THPA**C**HVSHPEL**C** *	α3β2 (41.2(22.5–75.5))				
Mr1.7[P1A]	***A***E**CC**THPA**C**HVSHPEL**C** *	α3β2 (42.0(26.1–67.6))				
Mr1.7[E2A]	P***A*CC**THPA**C**HVSHPEL**C** *	α3β2 (11.8(8.4–16.7)), α9α10 (>10,000), α3β4 (>10,000), α7 (>10,000), α2β4 (>10,000)	>847	>847	>847	>847
Mr1.7[H10A]	PE**CC**THPA**C*A***VSHPEL**C** *	α3β2 (123.4(96.5–157.6))				
Mr1.7[H13A]	PE**CC**THPA**C**HVS***A***PEL**C** *	α3β2 (>10,000)				
Mr1.7[V11G]	PE**CC**THPA**C**H***G***SHPEL**C** *	α3β2 (137.8(114.5–165.7))				
Mr1.7[S12N]	PE**CC**THPA**C**HV*N*HPEL**C** *	α3β2 (11.5(9.4–13.9)), α3β4 (849.9(477.4–1523.6)), α7 (220.3(136.4–355.9)), α2β4 (367.3(215.7–625.6)), α9α10 (>10,000)	74	32	17	>870
Mr1.7[H13N]	PE**CC**THPA**C**HVS***N***PEL**C** *	α3β2 (541.4(395.1–742.0)), α9α10 (1833(1033–3250)), α3β4 (>10000), α7 (2423(1785–3289)), α2β4 (>10,000), α4β2 (>10,000), α2β2 (>10,000), α4β4 (>10,000)	>18.5	>18.5	4.5	3.5
Mr1.7[E2A,S12N]	P***A*CC**THPA**C**HV***N***HPEL**C** *	α3β2 (6.4(5.1–7.9)), α3β4 (556.3(385.4–803.1)), α7 (590.8(447.0–781.0)), α2β4 (3489(2503–4863)), α9α10 (>10,000)	88	594	109	>1563
Mr1.7[V11G,S12N]	PE**CC**THPA**C**H***GN***HPEL**C** *	α3β2 (28.4(24.8–32.5))				
Mr1.7[E2G,V11G,S12N,Δ1]	*G***CC**THPA**C**H***GN***HPEL**C** *	α3β2 (4.4(3.7–5.3)), α3β4 (124.9(80.9–192.7)), α2β4 (389.9(250.3–607.5)), α9α10 (>10,000), α7 (>10,000)	27	89	>2272	>2272

### 2.3. NMR Assignments and Structural Calculations of Mr1.7

We found a total of 17 spin systems for Mr1.7 in the “fingerprint” region of a 120-ms TOCSY spectrum (Supplementary Table S2 and Figure S2), which were verified in a relevant DQF-COSY spectrum. Most spin systems were shown except for the disappeared amide H of the first residue and Pro. The NOE connections of d_Ni-Ni+1_, d_αi-Ni+1_ and d_βi-Ni+1_ well identified the residue assignments (Supplementary Figure S3).

In the present study, we determined the solution structures of Mr1.7 using the same strategy [22,23]. Most NOESY cross peaks were assigned and integrated, and then they were put into the cycles of structure calculations using Cyana program. Two disulfide-bond constraints (Cys^3^-Cys^9^ and Cys^4^-Cys^17^), which were demonstrated by HPLC analysis of the two-step folding products, were used in the structure calculation. A total of 156 NOE-based distance restraints were used in the process of Mr1.7, of which 96 were derived from intraresidue NOEs, 42 from sequential backbone NOEs, 15 from medium-range NOEs, and three from long-range NOEs (Table 2, Supplementary Figure S3). The cross peaks of H_α(His6)_-H_δ(Pro7)_ and H_α(His13)_-H_δ(Pro14)_ were found in spectra, indicating that Pro^7^ and Pro^14^ were in *trans* conformation. In addition, five dihedral angle constraints referring to Thr^5^, His^6^, Ser^12^, His^13^ and Leu^16^ were used to give J coupling constants. Moreover, a pair of H-bond constraints (carboxyl O of Pro^7^ to amide H of His^10^) derived from H-D exchange results were adopted as well.

**Figure 2:**
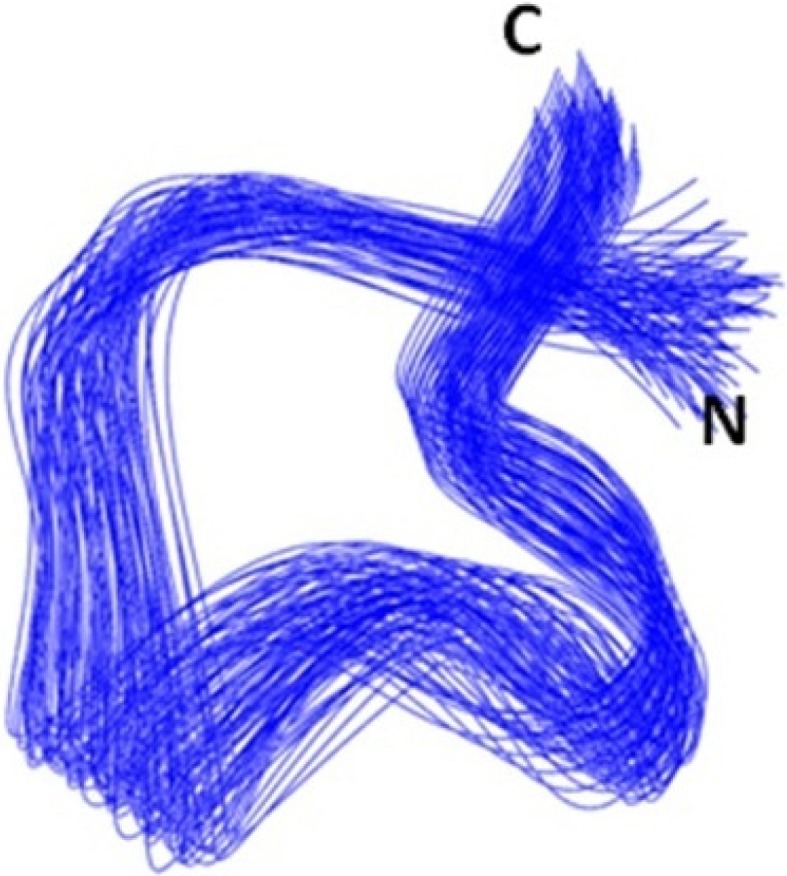
Backbone ensemble of 20 lowest energy structures of Mr1.7.

**Figure 3:**
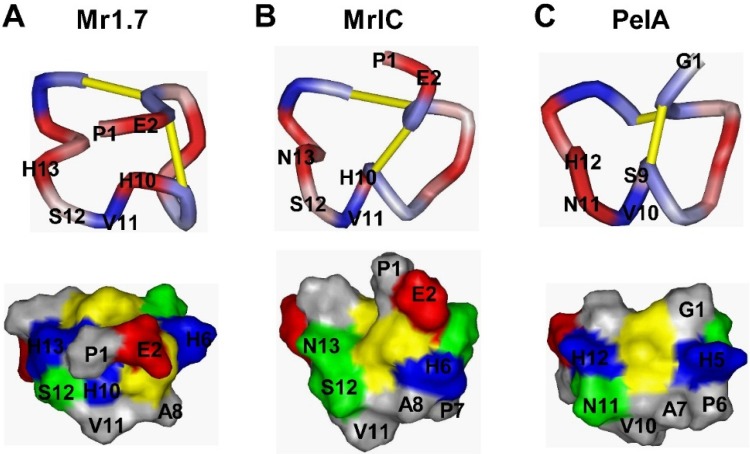
Comparison of the surfaces between Mr1.7, MrIC and PeIA. (**A**–**C**) represent the conformational comparison (top) and the distribution of the surface-exposed charge and polarity (bottom) of Mr1.7, MrIC and PeIA, respectively. Negatively and positively charged residues are shown in *red* and *blue*, respectively. Hydrophobic and hydrophilic residues are shown in *gray* and *green*, respectively. Cysteine residues are shown in *yellow*.

Figure 2 shows an overlay of the backbone atoms for the 20 structures of Mr1.7. The three-dimensional structure of Mr1.7 was characterized by a compact folding, based on the two disulfide bridges from Cys^3^-Cys^9^ and Cys^4^-Cys^17^. Similar to those of typical α-CTXs targeting nAChRs, the side chains of all residues oriented outside, making the whole conformation of Mr1.7 a typical ω twist. However, the secondary structure of Mr1.7, of which residues Pro^7^ to His^10^ were represented as a 3_10_-helix and the *C*-terminal sequence appeared a type I β-turn from residues Pro^14^ to Cys^17^, showed quite different from that of PeIA [24] and MrIC [25] (Figure 3 shows a surface representation of Mr1.7, MrIC and PeIA).

**Table 2:** Structural statistics of the ensemble of 20 structures of Mr1.7 after CYANA calculation.

Parameter	Value
**NOE distance constraints**	156
Intra-residue	96
Sequential	42
Medium range	15
Long range	3
**NMR constraint violations**	
H bond constraints	1
Dihedral constraints	5
**Cyana target function (Å)**	0.37 ± 0.05
**RMSD to mean coordinates**	
Mean global backbone atoms RMSD	0.66 ± 0.15
Mean global heavy atoms RMSD	1.22 ± 0.18
**Rachandran statistics from PROCHECK-NMR**	
Most favored regions, %	47.7
Additional allowed regions, %	43.1
Generously allowed regions, %	9.2
Disallowed regions, %	0.0

### 2.4. Potency of Mr1.7 at the Rat Neuronal nAChRs

**Figure 4:**
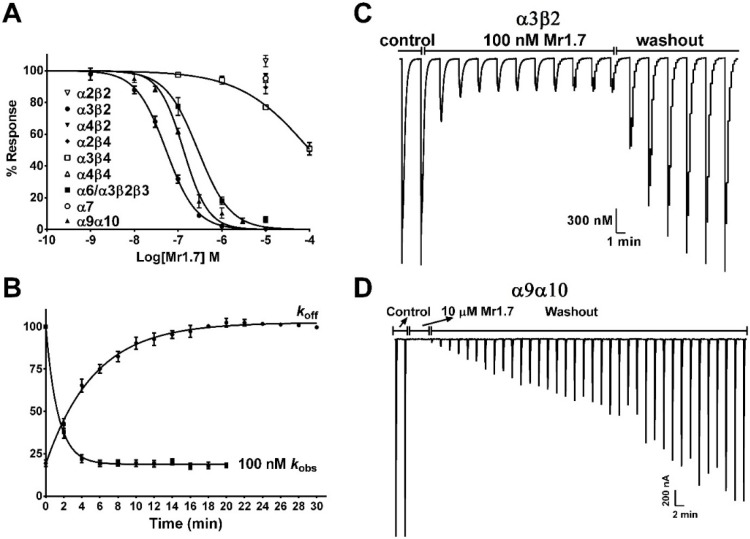
Effects of Mr1.7 on rat nAChRs expressed in *Xenopus* Oocytes. (**A**) Concentration-dependent response curves of Mr1.7 on various rat nAChR subtypes; (**B**) Kinetic analysis of the activity of Mr1.7 on nAChR α3β2. The data were fit to a single exponential equation; (**C**) A representative trace of 100 nM Mr1.7 was applied on nAChR α3β2; (**D**) Recovery from Mr1.7 block (10 μM) in nAChR α9α10. Peptides were applied by perfusion to oocytes expressing nAChRs as described in Materials and Methods. The error bars denote the S.E.M. of the data from four to nine oocytes for each determination. See Table 1 and Table 4 for a summary of the values obtained.

In the present study, we assessed the functional activity of Mr1.7 by activating ACh-evoked membrane currents in *Xenopus* oocytes. Figure 4A exhibits that it potently inhibited α3β2, α9α10 and α6/α3β2β3 subtypes with an IC_50_ of 53.1 nM, 185.7 nM and 284.2 nM (Table 1), respectively, but showed no inhibitory activity on other nAChR subtypes. The half time (*t*_1/2_) of the recovery of α3β2 subtype from its binding to 100 nM Mr1.7 was 3.710 (3.165–4.480) min (Figure 4B). Figure 4C shows a representative trace of ACh-evoked currents of α3β2 subtype inhibited by 100 nM Mr1.7. However, the recovery rate of Mr1.7 block for α9α10 was significantly slower compared with α3β2 subtype (Figure 4D).

### 2.5. Key Residues of the N-Terminal Sequence Affect the Potency and Selectivity of Mr1.7 for nAChR α3β2

**Figure 5:**
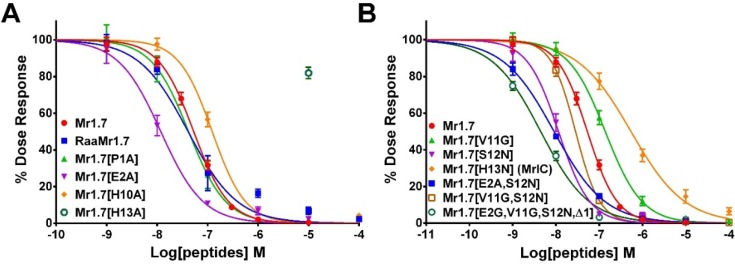
Effects of the key residues on the potency of Mr1.7 for rat nAChR α3β2. (**A**) The concentration-response analysis for the inhibition of α3β2 subtype by Mr1.7 and its Ala variants; (**B**) The concentration-response analysis for the inhibition of α3β2 subtype by Mr1.7 and its combinative variants. Peptides were applied by perfusion to oocytes expressing nAChRs as described in Materials and Methods. The error bars denote the S.E.M. of the data from four to nine oocytes for each determination. The values of IC_50_ on α3β2 subtype were summarized in Table 1.

**Figure 6:**
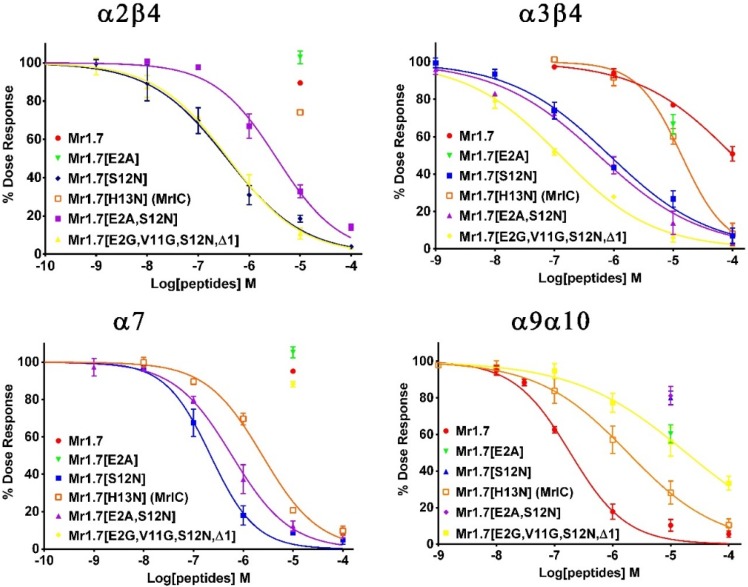
Concentration-response analysis of the activity of five potency variants of Mr1.7 on *Xenopus* oocyte-expressed nAChR α2β4, α3β4, α7 and α9α10 subtypes. The peptides were applied, as described in Materials and Methods, and the error bars for the data denote the S.E.M. from four to six oocytes for each determination. The IC_50_ values and 95% confidence intervals were summarized in Table 1.

To determine the effects of the *N*-terminal amino acid sequence of Mr1.7 for α3β2 subtype, we performed alanine mutation of PE residues ahead of the first Gly. The replacement of Pro^1^ by Ala resulted in a slight increase in the potency of Mr1.7, while the substitution of Glu^2^ with Ala led to a significant increase in the potency of Mr1.7 (Figure 5A). The IC_50_ of Mr1.7[E2A] was 11.8 nM and about five-fold lower than Mr1.7 (IC_50_ = 53.1 nM). In addition, the selectivity of Mr1.7[E2A] for α3β2 was significantly increased (SI > 847, Table 1) compared with α2β4, α3β4, α7 or α9α10 subtypes (Figure 6).

### 2.6. Key Residues of the Loop2 Region Affect the Potency and Selectivity of Mr1.7 for nAChR α3β2

The Ala variants of Mr1.7 or its combinative variants were synthesized and evaluated based on other typical α-CTXs with high potency and selectivity to α3β2 subtype (Table 3). Figure 5 and Table 1 revealed that the substitution of Val^11^ with Gly or the substitution of His^10^ with Ala resulted in the decrease in inhibitory activity, indicating that Val^11^ and His^10^ were the functional residues. In addition, Figure 5 also shows that the substitution of His^13^ with Ala and Asn resulted in more than 10-fold loss of inhibitory activity of Mr1.7, suggesting that a single substitution could generate great changes of activities. Interestingly, the variants containing the substitution of Ser^12^ to Asn substantially increased the potency (1–10-fold) for α3β2 subtype, and the IC_50_ of Mr1.7[S12N], Mr1.7[E2A,S12N] Mr1.7[V11G,S12N] and Mr1.7[E2G,V11G,S12N,Δ1] was 11.5, 6.4, 28.4 and 4.4 nM, respectively.

**Table 3:** Amino acid sequences of α-CTXs targeting nAChR α3β2. ^a^ Amino acid conservations are denoted by light gray shade; The scaffold formed by disulfide-bonded cysteines are in boldface and boxed; ^b^ all the targets are rat nAChRs unless otherwise indicated; h indicates human nAChRs; * *C*-terminal carboxamide; ^#^ *C*-terminal carboxylate; γ: γ-carboxyglutamate; O: 4-*trans*-hydroxyproline; Y: sulfated tyrosine.

α-CTX	Amino acids sequence ^a^	Targets (IC_50_, nM) ^b^	Reference
Mr1.7	PECCTHPACHVSHPELC *	α3β2 (53.1), α9α10 (187.5)	This work
Mr1.8 (MrIC)	PECCTHPACHVSNPELC *	α3β2 (541.4), α9α10 (1833), α7 (2423)	This work
PeIA	GCCSHPACSVNHPELC *	α3β2 (23), α9α10 (6.9), α3β4 (480), α7 (1800)	[26]
RegIIA	GCCSHPACNVNNPHIC *	α3β2 (33), α3β4 (97), α7 (103), α9α10 (>1000)	[27]
OmIA	GCCSHPACNVNNPHICG *	α3β2 (11.0), α7 (27.1)	[28]
LsIA	SGCCSNPACRVNNPNIC *	α3β2 (10.3), α7 (10.1)	[17]
ArIA	IRDECCSNPACRVNNOHVCRRR ^#^	α3β2 (18.0), α7 (6.0)	[29]
GID	IRDγCCSNPACRVNNOHVC ^#^	α3β2 (3.1), α4β2 (152), α7 (4.5)	[30]
ArIB	DECCSNPACRVNNPHVCRRR ^#^	α3β2 (60.1), α7 (1.8)	[29]
TxIA	GCCSRPPCIANNPDLC *	α3β2 (3.6), α7 (392)	[31]
PnIA	GCCSLPPCAANNPDYC *	α3β2 (9.6), α7 (252)	[32]
AnIA	CCSHPACAANNQDYC *	α3β2 (5.8)	[16]
AnIB	GGCCSHPACAANNQDYC *	α3β2 (0.3), α7 (76)	[16]
GIC	GCCSHPACAGNNQHIC *	hα3β2 (1.1), hα4β2 (309), hα3β4 (755)	[33]
Lo1a	EGCCSNPACRTNHPEVCD *	α7 (3240)	[34]
Vc1.1	GCCSDPRCNYDHPEIC *	α3β2 (5532), α9α10 (109), α3β4 (4200), α7 (7123)	[35]
MII	GCCSNPVCHLEHSNLC *	α3β2 (0.5), α7 (~200)	[36]
TxID	GCCSHPVCSAMSPI-C *	α3β4 (12.5), α2β4 (4550)	[37]
BuIA	GCCSTPPCAVLY---C *	α3β2 (5.7), α3β4 (28), α4β4 (69.9), α2β4 (121), α7 (272), α2β2 (800)	[38]

Moreover, we also assessed the selectivity of variants with high potency on other nAChR subtypes. Results showed that all above-mentioned variants harboring a substitution of Ser^12^ to Asn exhibited no activity on α2β2, α4β2 and α4β4 subtypes at a concentration of 10 μM. However, these variants exhibited a high potency for the α2β4, α3β4, α7 or α9α10 nAChR subtypes. For example, Mr1.7[S12N] simultaneously targeted α3β2, α3β4, α2β4 and α7 subtypes with an IC_50_ of 11.5 nM, 0.85 μM, 0.37 μM and 0.22 μM, respectively (Table 1). As a result, Mr1.7[S12N] lost its selectivity to nAChR subtypes. 

**Figure 7:**
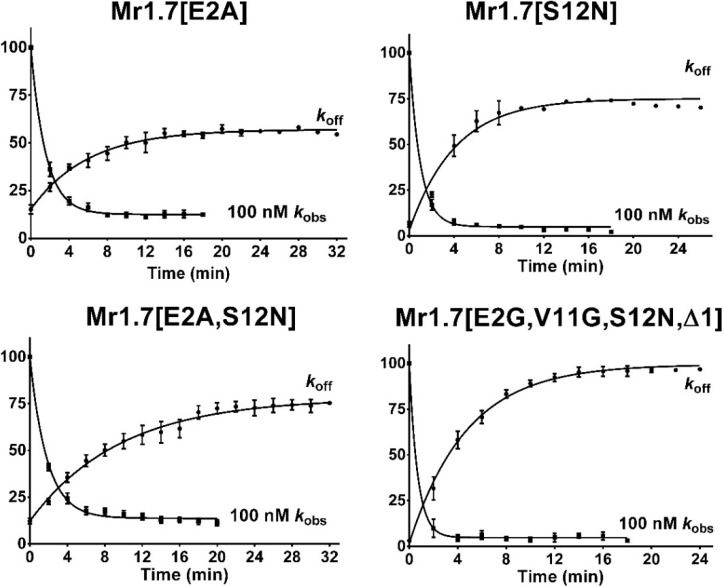
Kinetic analyses of Mr1.7 variants on *Xenopus* oocyte-expressed rat nAChR α3β2. The toxins were applied, as described under Materials and Methods, and the data were fit to a single exponential equation. The error bars denote the S.E.M. of the data from three to six oocytes for each determination. The kinetic data were summarized in Table 4.

We also performed the onrate and offrate kinetic experiments of the variants with a high potency on α3β2 subtype (Figure 7). The 50% recovery from block of Mr1.7[S12N] and Mr1.7[E2A,S12N] was 4.5 min and 5.9 min, respectively. However, the kinetic of Mr1.7[E2G,V11G,S12N,Δ1] was relatively rapid, with 50% recovery of 3.3 min. Table 4 summarizes the values obtained from the kinetic experiments.

In addition, according to the gene sequence of Mr1.7, an Arg may exist ahead of the *N*-terminal. Therefore, RaaMr1.7 was synthesized to determine its effect on nAChR α3β2. The results showed that RaaMr1.7 (IC_50_ = 41.2 nM) had a comparable inhibitory activity for α3β2 subtype with Mr1.7 (Figure 5A).

**Table 4:** Kinetic analysis of the block and recovery from block for nAChR α3β2 by Mr1.7 and its potency variants. ^a^ *t*_1/2_ = 0.693/*k*_off_; ^b^ *k*_obs_ = *k*_on_[toxin] + *k*_off_; ^c^ *K*_i_ = *k*_off_/*k*_on_; Data are means ± S.E.M. from three to six oocytes. Numbers in parentheses are 95% confidence intervals.

α-CTX	*k*_off_	*t*_1/2_^a^	*k*_obs_^b^	*k*_on_	*K*_i_^c^
	min^−1^	min	min^−1^	min^−1^M^−1^	M^−9^
Mr1.7	0.187 ± 0.016	3.710 (3.165–4.480)	0.735 ± 0.064	0.548 × 10^7^	34.093
Mr1.7[E2A]	0.168 ± 0.023	4.129 (3.236–5.702)	0.686 ± 0.040	0.518 × 10^7^	32.401
Mr1.7[S12N]	0.244 ± 0.038	2.836 (2.150–4.164)	1.024 ± 0.073	0.780 × 10^7^	31.349
Mr1.7[E2A,S12N]	0.104 ± 0.014	6.688 (5.263–9.170)	0.546 ± 0.031	0.443 × 10^7^	23.402
Mr1.7[E2G,V11G,S12N,Δ1]	0.211 ± 0.017	3.286 (2.842–3.894)	1.460 ± 0.2516	0.125 × 10^8^	16.884

## 3. Discussion

Up till now, several α-CTXs have been found to specifically target nAChR α3β2, including PeIA, MII, GIC, GID, Vc1.1, and so on (Table 3). They are all 4/7-α-CTXs and share similar amino acid residues in the loop1 region. The difference in selectivity is mainly derived from the surface-exposed charge and polarity of the loop2 region (Table 3). Most of these α-CTXs target α3β2 and α7 subtypes, and others also act on α3β4 and α2β4 subtypes. However, Mr1.7 mainly targeted the α3β2 (IC_50_ = 53.1 nM) and α9α10 nAChRs (IC_50_ = 187.5 nM), and the selectivity index (SI) of α3β2 to α9α10 is 3.5. Of all the α4/7-CTXs currently identified, only PeIA and Vc1.1 act on α3β2 and α9α10 subtypes (Table 3), while the latter two α-CTXs display a higher selectivity to α9α10 [26]. Although the primary structure of PeIA is highly homologous to Mr1.7 in loop1 and loop2 regions, their secondary structures are quite different [24]. In addition, the PE residues were located on the outer surface of Mr1.7, affecting the charge distributions (Figure 3A,C). As a result, the difference of selectivity of Mr1.7 might be derived from the Pro^1^ and Glu^2^ residues ahead of the first Cys.

We further confirmed the contribution of the above PE residues to potency and selectivity by the Ala variants of Mr1.7 at the *N*-terminal sequence. The results showed that the replacement of Glu^2^ by Ala significantly increased the potency and selectivity of Mr1.7 for α3β2 (Table 1), and the IC_50_ of Mr1.7[E2A] was four-fold higher than that of Mr1.7, showing no inhibitory activity on other nAChR subtypes (SI > 847). However, the substitution of Pro^1^ with Ala only led to a slight increase in potency. These results were consistent with the effects of *N*-terminal of MrIC, a Mr1.7 variant (Mr1.7[H13N]), which was recently reported to act as an agonist of the endogenous human α7 nAChR and a weak antagonist of human α7 nAChR expressed in *Xenopus* oocyte [25,39]. Actually, we firstly cloned MrIC by constructing cDNA libraries in 2012 and named it as Mr1.8 (the accession number of Mr1.8 is JF460791) [21]. We also found that MrIC was a weak antagonist of the rat nAChR subtypes (Table 3).

Several residues were mutated in the loop2 region based on the amino acid sequences of other α-CTXs in order to elevate the binding activity of Mr1.7 for α3β2 subtype. Firstly, the mutation of Val^11^ by Gly was performed because a shorter side-chain at this position has been reported to be better for the elevation of potency for β2-containing nAChRs [40]. However, Mr1.7[V11G] exhibited a lower potency than Mr1.7 for α3β2 subtype, suggesting that the side-chain at this position played other roles in the binding of Mr1.7 with α3β2 subtype. Secondly, we also assessed the role of residues at position 12 since several α-CTXs contain an Asn at this position, such as PeIA [41], RegIIA [27], PnIA [40], AnIA [16], AnIB [16], GIC [33], TxIA [31] and OmIA [28], displaying a high potency on α3β2 subtype (Table 3). Figure 5B and Table 1 showed that Mr1.7[S12N], Mr1.7[E2A,S12N], Mr1.7[V11G,S12N] and Mr1.7[E2A,V11G,S12N,Δ1] significantly increased the potency for the α3β2 nAChR, which was consistent with previous findings. However, the substitution of Ser^12^ with Asn resulted in the loss of selectivity to the α3β2 nAChR (Figure 5, Table 1). Interestingly, Mr1.7[S12N] and Mr1.7[E2A,V11G,S12N,Δ1] potently inhibited nAChR α2β4 with an IC_50_ of 367 nM and 390 nM, respectively. To date, it has been reported that two α-CTXs (BuIA and TxID) target the α2β4 subtype with an IC_50_ of 121 nM and 4550 nM, respectively [37,38]. Mr1.7[S12N] was the second potent α2β4 inhibitor, though its selectivity remains not high. 

Although the sequential difference between Mr1.7 and MrIC (Mr1.7[H13N]) is just a substitution at position 13, the secondary structure and the surface charges of Mr1.7 are quite different from those of MrIC and PeIA. The residue His^13^ in Mr1.7 helps form the turn near *C*-terminal and affects the entire structure. In the contrary, the secondary structure of MrIC is similar to PeIA (Figure 3) though the sequence was different. These differences in structures result in a great change of inhibitory activities.

It should be pointed out that Mr1.7 and its variants possessed a mediate recovery rate (*k*_off_ = 0.10~0.24 min^−1^, Table 4), which was similar to MII (*k*_off_ = 0.11 min^−1^) [42], but slower than BuIA (*k*_off_ = 0.656 min^−1^) [42]. Moreover, Mr1.7[S12N] and Mr1.7[E2A,V11G,S12N,Δ1] exhibited a lower *K*_i_, which was consistent with their high potency (Table 1).

## 4. Materials and Methods 

### 4.1. Peptide Synthesis

Mr1.7 and its variants were synthesized using a previously described method [43]. Briefly, the protected-peptide was synthesized and then cleaved from Rink resin with the cleavage solution (trifluoroacetic acid (TFA), 8.8 mL; water, 0.5 mL; DTT, 0.5 g; triisopropylsilane, 0.2 mL). The released peptides were oxidized in 0.1 M NH_4_HCO_3_ at room temperature, pH 8.0~8.2. The folded products were then purified and assessed using analytical reversed-phase HPLC. Table 1 lists the primary sequences of Mr1.7 and its variants.

### 4.2. Analysis of Disulfide Bridges

Because of the limited quantity of natural peptides, the disulfide arrangement of synthetic Mr1.7 by the one-step oxidative folding was determined through comparison of peptide folding products with known disulfide connectivity. The linear peptide containing an acetamidomethyl (Acm)-protecting group at the Cys (II-IV) position was folded by incubation in 0.1 M NH_4_HCO_3_ buffer (pH = 8.0) at room temperature for 24 to 36 h. A peptide with the different disulfide bridges (I-III, II-IV) was generated by further oxidizing the folded product with an iodine mixture containing 30% CH_3_CN, 2% TFA and 68% H_2_O for 10 min. This secondary oxidized product was co-applied with the one-step folding product, Mr1.7, onto an analytical C18 column.

### 4.3. NMR Spectroscopy and Structural Calculation

Samples of CTX Mr1.7 were prepared by dissolving peptides into 500 μL of either 9:1 (v/v) H_2_O/D_2_O or 99.99% D_2_O (Cambridge Isotope Lab, Andover, MA, USA) containing 0.01% TFA at pH 3.0. The final peptide concentration was approximately 3.0 mM. NMR spectra were collected on Bruker Avance 400 and 600 MHz NMR spectrometers at 298 K. The homonuclear DQF-COSY, TOCSY and NOESY spectra were obtained in a phase-sensitive mode using time-proportional phase incrementation for quadrature detection in the t1 dimension. Presaturation during the relaxation delay period was used to suppress the solvent resonance, unless specified otherwise. NOESY spectra were obtained with a mixing time of 300 ms. TOCSY spectra were collected using the MLEV-17 pulse scheme for a spin lock of 120 ms. In order to identify the slow exchange of backbone amide protons, each sample lyophilized from the hydrogen-containing solution was re-dissolved in a deuterium-containing solution. All chemical shifts were referenced to the methyl resonance of 4,4-dimethyl-4-silapentane-1-sulfonic acid (DSS) used as internal standard. The spectra were processed using Bruker Topspin 2.1 and analyzed by Sparky 3.1 [44].

Structural calculations were performed with distance constraints derived from the NOESY spectra of Mr1.7 using CYANA 2.1 software [45]. Dihedral angle restraints were determined based on the *^3^J_HN-Ha_* coupling constants derived from the DQF-COSY spectral analysis. The φ angle constraints for some residues were set to −120 ± 40° for *^3^J_HN-Ha_* > 8.0 Hz and −65 ± 25° for *^3^J_HN-Ha_* < 5.5 Hz, respectively. In addition, backbone dihedral constraints were not applied for *^3^J_HN-Ha_* values ranging from 5.5 Hz to 8.0 Hz. Based on the slow exchange of amide protons in hydrogen-deuterium exchange experiments, the hydrogen bond constraints were added as target values of 2.2 Å and 3.2 Å for the NH(i)–O(j) and N(i)–O(j) bonds, respectively. The 20 lowest energy conformers were submitted to a molecular dynamics refinement procedure using the Sander module of the Amber 9 program. The final outcomes were used for structural quality analysis using MOLMOL software, and the geometric qualities of the refined structures were evaluated using PROCHECK-NMR software. The data, including chemical shifts, were submitted to the BMRB database with access code 19639 for Mr1.7.

### 4.4. Inhibitory Activity to nAChR Subunits Expressed on Oocyte

Briefly, cRNA preparation, oocyte harvesting and expression of nAChR subunits were performed as previously described [23,36]. The *Xenopus* oocytes were incubated in ND96 solution (96.0 mM NaCl, 2.0 mM KCl, 1.8 mM CaCl_2_, 1.0 mM MgCl_2_ and 5 mM HEPES, pH~7.3) containing 2.5 mM pyruvic acid sodium (Sigma, St. Louis, MO, USA) and antibiotics (100 U/mL penicillin, 100 mg/mL streptomycin, Sigma) at 18 °C. Recordings were performed 2–5 days post-injection at room temperature (~22 °C).

The oocyte was continuously gravity-perfused with 5 min intervals until peak current amplitude was obtained. For the dose response, the oocyte was perfused with toxin solution until equilibrated (5–10 min). In high-dose experiments (1 μM or greater), 5.5 μL of a 10-fold concentrated toxin solution was directly pipetted into static bath 5 min prior to the exposure of ACh pulses.

Association and dissociation rate constants were calculated from single exponential equation (Y = Y_max_ × (exp(−*k*_off_ × t)) for dissociation and (Y = Y_max_ × (1 − exp(−*k*_obs_ × t)) for association, where Y_max_ is bound ligand at equilibrium. The dose-response data were fit to the equation: % response = 100/[1 + ([toxin]/IC_50_)*^n^*], where *n* is the Hill coefficient and IC_50_ is the antagonist concentration giving half-maximal response, by non-linear regression analysis using GraphPad Prism (GraphPad Software, San Diego, CA, USA).

## 5. Conclusions

In summary, Mr1.7 specifically inhibited α3β2, α9α10 and α6/α3β2β3 nAChRs, the PE residues at the *N*-terminal sequence of Mr1.7 were important for modulating its binding activity and selectivity, and the combinative mutations in the *N*-terminal and loop2 significantly increased the binding of Mr1.7 to nAChR subtypes. Taken together, our work expanded our knowledge of selectivity and provided a new way to improve the potency and selectivity for nAChR subtypes.

## Supplementary Files

Supplementary File 1
