# Supplementary material for: Structural and Functional Characterization of a Novel α-Conotoxin Mr1.7 from Conus marmoreus Targeting Neuronal nAChR α3β2, α9α10 and α6/α3β2β3 Subtypes
Source: Mar Drugs. 2015 May 27;13(6):3259–75. doi: 10.3390/md13063259 (PMC4483627; doi:10.3390/md13063259)
Supplement: Supplementary File 1 [file marinedrugs-13-03259-s001.pdf]

Supplementary Information

Table S1. Molecular mass of Mr1.7 and its variants. * *C*-terminal amidated; The mutated residues and Cysteines are in bold and italic face.

α-CTX	Amino Acid Sequence	Theoretical MW (Da)	*m/z* (+1)
Mr1.7	PE**CC**THPA**C**HVSHPEL**C** *	1856.72	1857.77
RaaMr1.7	***R***PE**CC**THPA**C**HVSHPEL**C** *	2012.85	2013.86
Mr1.7[P1A]	***A***E**CC**THPA**C**HVSHPEL**C** *	1830.74	1831.70
Mr1.7[E2A]	P***A*****CC**THPA**C**HVSHPEL**C** *	1798.75	1799.75
Mr1.7[H10A]	PE**CC**THPA**C*****A***VSHPEL**C** *	1790.73	1791.71
Mr1.7[V11G]	PE**CC**THPA**C**H***G***SHPEL**C** *	1814.71	1815.75
Mr1.7[S12N]	PE**CC**THPA**C**HV***N***HPEL**C** *	1883.76	1884.76
Mr1.7[V11G,S12N]	PE**CC**THPA**C**H***GN***HPEL**C** *	1841.72	1842.67
Mr1.7[E2A,S12N]	P***A*****CC**THPA**C**HV***N***HPEL**C** *	1825.73	1826.76
Mr1.7[E2G,V11G,S12N,Δ1]	***G*****CC**THPA**C**H***GN***HPEL**C** *	1672.61	1673.74

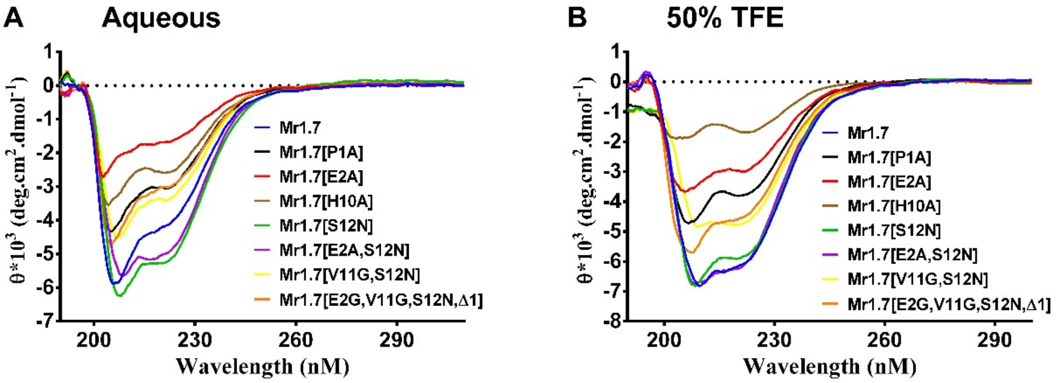

Figure S1. Circular dichroism spectra of Mr1.7 and its variants. (**A**) CD of the peptides in 0.01 M PBS (pH = 7.2); (**B**) CD of the peptides in 0.01 M PBS and 50% TFE (pH = 7.2). Circular dichroism (CD) spectra were measured between 190 and 340 nm on a BioLogic Mos 450 spectropolarimeter (Grenoble, France). The peptides were dissolved in 0.01 M phosphate buffer (pH 7.2) or 0.01 M phosphate buffer containing 50% trifluoroethanol (TFE) to a final concentration of 35 μM. A 1 cm path length quartz cell was employed. Each spectrum represents the accumulation of eight individual scans collected at 1.0 nm intervals at a bandwidth of 1.0 nm. The CD spectra indicated Mr1.7 variants had similar α-helical structure with that of Mr1.7, suggesting they had the same disulfide connectivity (I-III, II-IV).

Table S2. Chemical shift assignments of Mr1.7.

Residue	HN	α	β	Other
Pro1	-	4.42	2.49	γ2.02 δ3.43
Glu2	9.09	4.29	2.12	γ2.49
Cys3	8.88	4.56	3.26, 2.50	-
Cys4	8.00	4.30	3.31, 2.95	-
Thr5	7.81	4.39	4.31	γ 1.17
His6	8.06	5.32	3.40	δ7.39 ε8.63
Pro7	-	4.30	2.42, 2.22	γ2.04 δ4.12, 3.98
Ala8	8.68	4.20	1.43	-
Cys9	7.65	4.22	3.31	-
His10	8.82	4.39	3.55, 3.32	δ7.24 ε8.73
Val11	8.28	3.97	2.21	γ1.06, 1.00
Ser12	7.45	4.45	3.98, 3.82	-
His13	7.67	5.18	3.32, 3.12	δ7.44 ε8.66
Pro14	-	4.36	2.35	γ2.05 δ3.62, 3.46
Glu15	8.56	4.27	2.12	γ2.57, 2.50
Leu16	7.63	4.32	1.79	γ1.51 δ0.91, 0.80
Cys17	7.73	4.79	2.58, 2.38	-

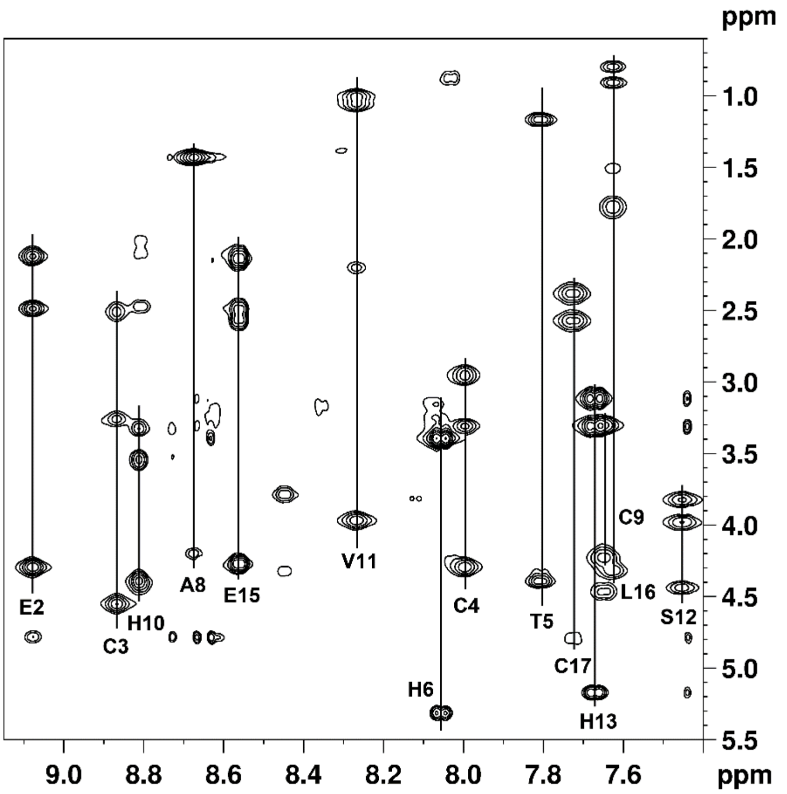

Figure S2. The portion of TOCSY spectrum of Mr1.7.

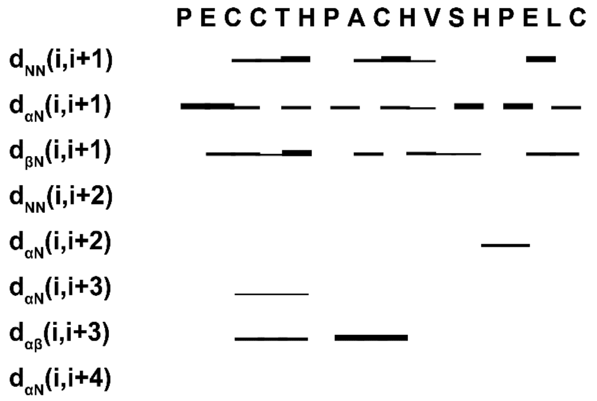

Figure S3. Summary of the sequential NOE connectivities involving the H_N, H_α, and H_β protons measured at 298 K for Mr1.7.

© 2015 by the authors; licensee MDPI, Basel, Switzerland. This article is an open access article distributed under the terms and conditions of the Creative Commons Attribution license (http://creativecommons.org/licenses/by/4.0/).
